# Lead Management Consensus in 2018: From Recommendations to Research

**DOI:** 10.19102/icrm.2018.091205

**Published:** 2018-12-15

**Authors:** Jay A. Montgomery, Christopher R. Ellis

**Affiliations:** ^1^Vanderbilt Heart and Vascular Institute, Nashville, TN, USA

**Keywords:** Extraction, lead management, lead removal

While 2017 brought a new consensus document from the Heart Rhythm Society (HRS) on cardiovascular implantable electronic device (CIED) lead management and extraction,^1^ 2018 saw the smaller-in-scope European Heart Rhythm Association (EHRA) consensus statement on lead extraction provide recommendations on definitions, endpoints, research trial design, and data collection.^2^ These two papers serve two different purposes. The former exists primarily to provide recommendations for lead extraction indications and procedural setup and offers guidance for individual leads that may require special consideration or equipment. Shared decision-making, operator training, leadless pacemakers, and the management of associated complication are addressed as well. In addition, five real-world scenarios are put forward with key points that serve to highlight not only the indications for extraction but also examples of settings in which lead abandonment may be the right choice to make. The document additionally contains management recommendations including class I recommendations for extraction in most scenarios in which it is certain or very likely that a CIED system is infected or when valvular endocarditis or persistent or recurrent bacteremia and fungemia are present. Most other scenarios, with the exception of superior vena cava syndrome, receive a class IIa or class IIb recommendation, with a focus on the primacy of patient factors and patient preferences (eg, magnetic resonance imaging conditionality, leadless devices, avoiding longer-term risks of abandoned leads).

Interestingly, while the 2017 HRS document only briefly comments on definitions, data collection, and research, the 2018 EHRA document focuses almost entirely on these things. Where the two documents overlap, there is no significant disagreement, which is not surprising given that the EHRA document is endorsed by the HRS. A few more minor additions to the definitions added in the EHRA document, however, include:

- Lead function refers to any lead function, including pacing, sensing, and/or defibrillation
- Lead failure refers to the loss of any lead function
- The EHRA document spells out the difference between class I, class II, and class III recalls according to the United States Food and Drug Administration versus the European Medicines Agency
- The EHRA document uses the term “inferior approach” to refer to leads extracted by what the HRS document refers to as a “femoral approach”
- The EHRA defines the term “surgical approach” to refer to extraction via sternotomy, minithoracotomy, or hybrid approach

Both documents make the case that centers performing lead extraction should be collecting data in a standardized way, using the definitions provided by these documents. The EHRA document spells out specific data collection fields and suggests the use of the Research Electronic Data Capture (https://projectredcap.org/; National Institutes of Health, Bethesda, MD, USA) resource as a potential multisite platform. This is precisely the process used at our institution with the Vanderbilt Transvenous Lead Extraction Registry (Va-TLER).

While there is significant overlap, these two consensus statements serve individually to provide guidance for clinical management as well as data collection and standardization. For the clinician struggling with a difficult lead management conundrum, the HRS 2017 document is an invaluable resource, giving class I, class IIa, and class IIb guidelines for specific scenarios and, perhaps more importantly, providing five real-world scenarios with key points that serve to highlight not only the indications for extraction but also scenarios where lead abandonment may be appropriate. Conversely, for the clinical researcher or quality-control analyst looking to establish a lead extraction registry, the EHRA 2018 document provides excellent guidance and standardization of definitions and categories of lead failure and extraction approaches.

## References

[1] Kusumoto FM, Schoenfeld MH, Wilkoff BL (2017). 2017 HRS expert consensus statement on cardiovascular implantable electronic device lead management and extraction.. Heart Rhythm..

[2] Bongiorni MG, Burri H, Deharo JC (2018). 2018 EHRA expert consensus statement on lead extraction: recommendations on definitions, endpoints, research trial design, and data collection requirements for clinical scientific studies and registries: endorsed by APHRS/HRS/LAHRS.. Europace..

